# DNA non-homologous end-joining enters the resection arena

**DOI:** 10.18632/oncotarget.22075

**Published:** 2017-10-26

**Authors:** Penny A. Jeggo, Markus Löbrich

**Affiliations:** Markus Löbrich: Darmstadt University of Technology, Radiation Biology and DNA Repair, Darmstadt, Germany

**Keywords:** DNA double-strand breaks, Ionizing radiation, non-homologous end-joining, resection, deletions

DNA double-strand breaks (DSBs) are arguably the most severe genetic damages that threaten cellular viability. They occur physiologically during recombinational processes to generate antibody diversity in immune cells or genetic variability in germ cells. They also arise following exposure to exogenous agents such as ionizing radiation or chemotherapeutic drugs. Understanding how cells respond to DSBs lies at the heart of evaluating the efficacy of radio- and chemotherapy as well as assessing risks from low dose radiation exposure [1].

The DNA damage response (DDR) pathways comprise mechanisms to halt cell cycle progression, pathways to repair the damage and routes to activate cell death, such as apoptosis. Two conceptually different repair pathways counteract the presence of DSBs, homologous recombination (HR) and canonical non-homologous end-joining (c-NHEJ).The former process elegantly repairs DSBs using an identical copy on the sister chromatid, and thus can restore any genetic information lost at the DSB site. This copying mechanism requires invasion by a single strand of the damaged chromosome to base-pair with the same sequence on the sister chromatid. The process for generating single-stranded tails at DSBs is called end-resection and is argued to determine whether DSB repair occurs by HR or c-NHEJ [2].

c-NHEJ, in contrast, does not have the capacity to retrieve genetic information lost at the break site. Instead, this pathway mends the two ends of a DSB without the need for sequence homology (hence the name “non-homologous end-joining”). The beauty of c-NHEJ is its simplicity, being fast, versatile and able to function in all cell cycle stages. Hence, it is the major pathway repairing exogenously induced DSBs in pre-replicative cell cycle phases; HR, in contrast, is restricted to post-replicative cell cycle phases where a sister homologue is available [3]. A fundamental difference in the initiation of the two pathways is end-resection, which is believed to promote HR and suppress c-NHEJ [2].

However, recent work has challenged the dogma that end-resection suppresses c-NHEJ and shown that a sub-pathway of c-NHEJ involves an orchestrated resection process [4]. Although alternative NHEJ pathways that handle resected DSBs have previously been described, such alternative modes of DSB repair mainly occur in the absence of the two classical DSB repair pathways [5].In contrast, the sub-pathway described in Biehs et al. [4] is used in conjunction with resection-independent c-NHEJ. Although factors regulating the interplay between the two forms of c-NHEJ remain unclear, resection-dependent c-NHEJ appears to be used at DSBs that arise in “challenging” genomic regions or at DSBs harbouring complex end-structures. Importantly, resection-dependent c-NHEJ represents the pathway that repairs 10-20% of the DSBs induced by ionising radiation with slow kinetics in G1-phase cells, a process initially unearthed using physical methods to monitor DSB repair and more recently dissected using current “DDR foci” approaches [6]*.* Most notably, the resection process leading to c-NHEJ uses many of the same factors and enzymes as the resection process in the context of HR, although several fundamental differences exist which tailor the resection process to either c-NHEJ or HR [4]. Importantly, resection during c-NHEJ is generally more limited, a feature that is likely necessary for compatibility with the c-NHEJ machinery which has evolved to fix double-stranded and not single-stranded DNA ends. This concept is also pursued by the mode of initiating resection, starting from the end and maintaining the end-joining machinery at the break site for resection-dependent c-NHEJ and, for HR, initiating resection internally followed by eviction of the end-joining machinery and subsequent generation of long stretches of single-stranded DNA. Another difference is that the process initiating resection is regulated by cell stage specific kinases, PLK3 in G1 for c-NHEJ and CDKs in S/G2 for HR [7]. Finally, it is noteworthy that failure to initiate resection in either phase can allow repair by resection-independent c-NHEJ whilst failure to block downstream steps confers a DSB repair defect, a feature that has long obscured the identification of resection-dependent c-NHEJ.

An interesting but yet unanswered question concerns the fidelity of repair. Whereas resection pursued by HR will eventually restore the original sequence information at the break site, the fidelity of resection-dependent c-NHEJ is currently unclear. The absence of an intact template would suggest that resection-dependent c-NHEJ would predominantly confer small deletions. It is possible that the generation of resected ends provides a signal which halts cell cycle progression allowing the completion of repair before the onset of replication - similar to the way in which resected ends during HR provide a checkpoint signal. In this case, resection-dependent NHEJ might be regarded as a last-resort pathway which ensures that even the most difficult DSBs are repaired before replication commences, albeit at the cost of generating small deletions.

However, an alternative explanation is that RNA molecules, generated by the significant proportion of our genome that is transcribed, might be exploited to retrieve sequence information lost at the DSB site. Indeed, there is increasing evidence that RNA is observed at DSB sites [8]. Further studies are required to address the important issue of the fidelity of DSB repair, particularly in the light of the findings that a significant number of DSBs are repaired via a resection-mediated pathway in the absence of a sister chromatid as a template.
